# Analysis of the Antenna Array Orientation Performance of the Interferometric Microwave Radiometer (IMR) Onboard the Chinese Ocean Salinity Satellite

**DOI:** 10.3390/s20185396

**Published:** 2020-09-21

**Authors:** Yan Li, Mingsen Lin, Xiaobin Yin, Wu Zhou

**Affiliations:** 1 Piesat Information Technology Co., Ltd., Beijing 100195, China; liyan_hy@piesat.cn (Y.L.); yinxiaobin@piesat.cn (X.Y.); 2 Key Laboratory of Space Ocean Remote Sensing and Application, Ministry of Natural Resources, Beijing 100081, China; zhouwu@mail.nsoas.org.cn; 3 Southern Marine Science and Engineering Guangdong Laboratory (Guangzhou), Guangzhou 511458, China; 4 National Satellite Ocean Application Service, Beijing 100081, China

**Keywords:** Chinese Ocean Salinity Satellite, Interferometric Microwave Radiometer, sea surface salinity, brightness temperature, antenna array orientation

## Abstract

The Chinese Ocean Salinity Satellite is designed to monitor global sea-surface salinity (SSS). One of the main payloads onboard the Chinese Ocean Salinity Satellite, named the Interferometric Microwave Radiometer (IMR), is a two-dimensional interferometric radiometer system with an L-band, Y-shaped antenna array. The comparison of two different array orientations is analyzed by an end-to-end simulation based on the configuration of the IMR. Simulation results of the different array orientations are presented and analyzed, including the brightness temperature (TB) images, the distribution of the incidence angles in the field of view, the TB radiometric resolutions, the spatial resolutions, the number of measurements in the Earth grid and the expected SSS accuracy. From the simulations we conclude that one of the array orientations has better performance for SSS inversion than the other one. The advantages mainly result in wider swath and better SSS accuracy at the edge of the swath, which then improve the accuracy of the monthly SSS after averaging. The differences of the Sun’s effects for two different array orientations are also presented. The analysis in this paper provides the guidance and reference for the in-orbit design of the array orientation for the IMR.

## 1. Introduction

Ocean salinity is a key ocean dynamic parameter that critically contributes to density-driven global ocean circulation and the Earth’s climate [1]. Knowing the salinity distribution at global scale and its annual and interannual variability is crucial to better understand the ocean’s role in the climate system, regulated by this circulation and water and heat fluxes between atmosphere and ocean [2,3,4]. The L-band microwave radiometer has been widely agreed as the most effective tool to measure sea-surface salinity (SSS) from space. However, the retrieval of SSS is still challenging in the L-band due to the low sensitivity of brightness temperature (TB) to SSS (from 0.8 K down to 0.2 K per psu depending on sea surface temperature, incidence angle, and polarization [5]).

Three L-band satellite missions have been launched, including European Space Agency (ESA)’s Soil Moisture Ocean Salinity mission (SMOS), National Aeronautics and Space Administration (NASA) and Comisión Nacional de Actividades Espaciales (CONAE)’s Aquarius/SAC-D mission and NASA’s Soil Moisture Active-Passive missions (SMAP). It appears that two diverse but suitable approaches are identified to be L-band microwave radiometry, while using interferometric system like the SMOS, and using large-size real aperture antenna regarding the Aquarius/SAC-D and the SMAP [6]. SMOS, operated since the end of 2009, was the first space-borne interferometric radiometer system that addressed the global measurements of SSS. The Microwave Imaging Radiometer with Aperture Synthesis (MIRAS), the only payload onboard SMOS, was also the first L-band radiometer that adopted interferometry technology in space [7,8]. Aquarius and SMAP both adopted a real aperture system but with different antenna types, and had the ability of combined active and passive measurements. Aquarius was equipped with a moderate sized reflector antenna (about 2.5 m in diameter) with the push broom mode, mainly dedicated to SSS measurement. The active device on Aquarius was a L-band scatterometer used for the sea surface roughness correction [9,10]. The other mission, SMAP, had a relatively large deployable mesh reflector antenna (about 6 m in diameter). The design of a L-band radiometer with a L-band radar enabled SMAP to observe soil moisture with high spatial resolution [11]. Unfortunately, Aquarius stopped operating on 7 June 2015 due to hardware failure, and SMAP also lost its radar instrument in mid 2015 due to an anomaly in the high-power amplifier [6].

China put forward a new ocean salinity satellite mission, which is designed to fill the vacancy of SSS monitoring capability in the Chinese ocean dynamic satellite series. The Chinese Ocean Salinity Satellite equips with two main payloads, named the Interferometric Microwave Radiometer (IMR) and the Microwave Imager Combined Active and Passive (MICAP) [12,13,14], which both adopt interferometric technology to implement L-band SSS observation. One of the payloads IMR is a two-dimensional (2D) instrument with an antenna array of Y shaped arrangement which is similar to SMOS’s MIRAS. The other payload, MICAP, is a one-dimensional (1D) array with L/C/K tri-band and the additional capability of active observation by an L-band scatterometer. The Chinese Ocean Salinity Satellite will provide important technical support for global ocean resource exploitation, disaster prevention, agriculture and environmental monitoring. At present, the Chinese Ocean Salinity Satellite has finished its project design and key technology study, and is heading into the engineering development stage.

This study focuses on the analysis of the array orientation impact and its effects on SSS retrieval through an end-to-end simulation system according to the configuration of the IMR. The analysis in this paper provides useful guidance and reference for the design and progress of the IMR. This paper is organized as follows: Section 2 provides a brief introduction for two payloads onboard Chinese Ocean Salinity Satellite and the antenna array of the IMR. The models and methods involved in the end-to-end simulation are described. The results for two different array orientations of the IMR, including the TB images, the incidence angles, the TB radiometric resolutions, the spatial resolutions, the numbers of the measurements in earth grid, the accuracy of expected SSS and the sun effects, are presented in Section 3. Finally, the conclusions and discussions are presented in Section 4.

## 2. Concepts and Methods

### 2.1. Payloads Onboard Chinese Ocean Salinity Satellite

**Figure 1:**
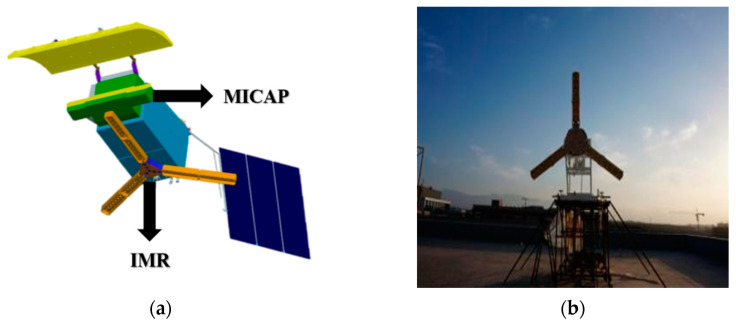
(**a**) Design of payloads Interferometric Microwave Radiometer (IMR) and Microwave Imager Combined Active and Passive (MICAP) onboard the Chinese Ocean Salinity Satellite, (**b**) prototype of the of IMR with the Y-shaped antenna array.

Both the IMR and the MICAP adopt interferometric technology to implement the need of large antenna size for the L-band radiometer. As an L-band triple polarized 2D interferometric microwave radiometer, the IMR is featured with the abilities of reasonable radiometric resolution, high spatial resolutions, and multiple incidences ability to observe SSS. The MICAP is a kind of active/passive instrument package, which includes L/C/K tri-band 1D interferometric radiometer and L-band digital beamforming scatterometer, sharing a parabolic cylinder reflector. As a 1D system, the MICAP has lower system complexity compared with the 2D system, which enable higher stability and accuracy in orbit. The C and K bands can be used to obtain real-time information of the sea surface temperature and the wind speed, and the scatterometer is used to correct the effect of sea surface roughness. The design of the Chinese Ocean Salinity Satellite with the configuration of two payloads are displayed in Figure 1a, and the prototype for the IMR is shown in Figure 1b.

### 2.2. Interferometric Microwave Radiometer’s (IMR) Y-Shaped Antenna Array

**Figure 2:**
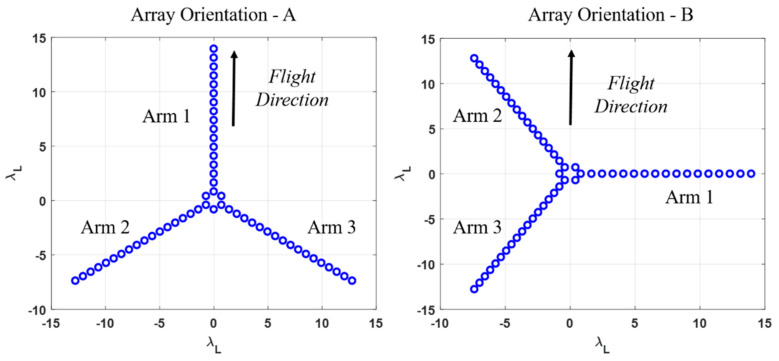
Arrangement of IMR’s antenna array with the spacing of antenna elements normalized to wave length of L-band ($\lambda_{L}$), and the three arms in the Y-shaped array are label as Arm 1, 2 and 3, respectively. Two different array orientations are named as A and B, and the flight direction are indicated as black arrows.

Compared with SMOS’s MIRAS, the IMR is a Y-shaped antenna array with 56 feed elements. The arrangement of the antenna array for the IMR is shown in Figure 2. The IMR’s Y-shaped antenna array is equipped with 3 arms which are equally spaced with an angular separation of 120° and are labeled as Arm 1, Arm 2 and Arm 3 (Figure 2). The total 56 elements are arranging as follow, 3 feed elements are placed in the center of the array and the rest of 53 are placed symmetrically in 3 arms, while Arm 1 contains 17 feed elements and the remaining arms each has 18 feed elements. The minimum antenna spacing of each feed element is 0.82 wave length of L-band (0.82$\lambda_{L}$). A comparison of designed characteristics and performance requirements for the Y-shaped instrument IMR and the MIRAS is summarized in Table 1.

In this paper, the simulation analysis is based on the two different orientations of the IMR’s antenna array which are named as array orientation A and array orientation B, as shown in Figure 2. Since the flight direction of the satellite in the simulation is fixed, the array orientation B is derived from the rotation of the array orientation A by 90° clockwise. The two different orientations of the antenna array have different performances in each step of the simulation from TB images to retrieved SSS.

### 2.3. End-to-End Simulation System

**Figure 3:**
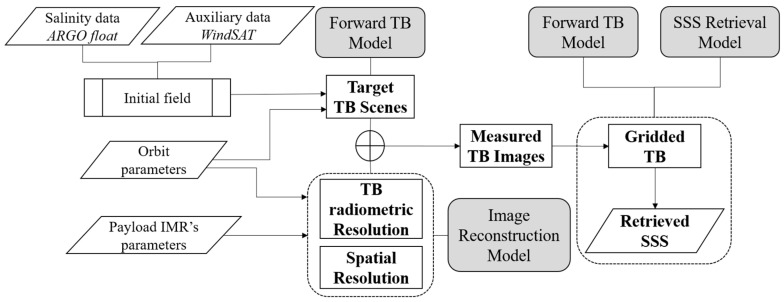
Diagram of the end-to-end simulation of sea-surface salinity (SSS) according to the configuration of the IMR, which consists of three major models highlighted as grey cases (i.e., the TB forward model, the image reconstruction model, and the SSS retrieval model).

An end-to-end simulation system is designed according to the configuration of the IMR. The end-to-end simulation consists of three main models: the TB forward model, the image reconstruction model and the SSS retrieval model (Figure 3). Some of the models used in this simulation are derived from previous work in [15].

The initial field data contain SSS data from Argo float [16] and auxiliary data from WindSat [17] which include sea surface temperature (SST), wind speed (WS) and atmospheric vapor. Both the SSS and other auxiliary data are monthly averaged maps with spatial resolution of 0.25°. The orbit used in this simulation is a classic sun-synchronous orbit. The orbit’s Kepler elements are set as follows: the semi-major axis is 6371 km, the inclination is 98°, the eccentricity is 0.0025 and the local time of descending node is 6:00 a.m.

First, the TB forward model produces target TB senses according to the parameters of the initial field and the orbit setting. Next, the TB radiometric resolution and the spatial resolution based on the configuration of the IMR are derived from the image reconstruction model. Then, IMR’s measured TB images simulated along the track with different distributions of the TB radiometric resolutions and the spatial resolutions are output from the image reconstruction model. Finally, retrieved SSS are iterated using the SSS retrieval model and the TB forward model, and the iteration is applied on gridded TBs which derived from the simulated TB images after projection into a pre-defined Earth grid.

The descriptions of the TB forward model, the image reconstruction model and the SSS retrieval model used in this end-to-end simulation are specified in the following subsections, respectively.

#### 2.3.1. Brightness Temperature (TB) Forward Model

The TB forward model produces TB from target scenes which are used as the input observations in the next simulation step and used in the parameter retrieval process also.

The sea surface TB $T_{B,sea}$ can be expressed as the sum of two terms, the TB in case of completely flat sea $T_{B,flat}$ and the additional TB due to the surface roughness $T_{B,rough}$ [18], as expressed in Equation (1).

(1) $$T_{B,sea}=T_{B,flat}+T_{B,rough}=\left(1-R_{fresnel}\right)\cdot SST+T_{B,rough}$$

where, $T_{B,flat}$ can be described as a function of the SSS, the SST and the Fresnel reflectivity $R_{fresnel}$. $T_{B,rough}$ refers to the TB contribution from the sea surface roughness [19,20].

$R_{fresnel}$ in Equation (1) depends on the incidence angle θ, the complex dielectric constant of sea water ε and the polarization [21]. The equations for $R_{fresnel}$ are shown in Equation (2).

(2) $$R_{fresnel,\;H}={\left|\frac{\cos\theta -\sqrt{\varepsilon -\sin^{2}\theta }}{\cos\theta +\sqrt{\varepsilon -\sin^{2}\theta }}\right|}^{2}\;R_{fresnel,\;V}={\left|\frac{\varepsilon \cos\theta -\sqrt{\varepsilon -\sin^{2}\theta }}{\varepsilon \cos\theta +\sqrt{\varepsilon -\sin^{2}\theta }}\right|}^{2}$$

where, $R_{fresnel,H}$ and $R_{fresnel,V}$ are the Fresnel reflectivity in horizontal polarization (H-pol) and vertical polarization (V-pol), respectively. ε is calculated with the Klein and Swift model [22].

Then, according to the radiative transfer model, the TB forward model at the top of the atmosphere in the Earth reference frame can be expressed in Equation (3):

(3) $$T_{B}=\left(T_{B,sea}+T_{DN}\cdot \Gamma +T_{Gal}\cdot \Gamma \cdot e^{-\tau_{atm}}\right)\cdot e^{-\tau_{atm}}+T_{UP}$$

where, $T_{DN}$ and $T_{UP}$ are the downward and upward atmospheric radiation. $e^{-\tau_{atm}}$ is the atmospheric attenuation and $\tau_{atm}$ is the atmospheric transmittance [23]. Γ is the sea surface reflection coefficient which can be expressed as $\Gamma =1-\left(T_{B,\;\mathrm{flat}}+T_{B,rough}\right)/SST$. $T_{GAL}$ is the cosmic and galactic contribution.

After the calculation of TB in the earth reference frame, TB in the antenna reference frame can be expressed in Equation (4) through multiplying the polarization rotation matrix.

(4) $$\left[\begin{array}{l} T_{A,X}\\ 2T_{A,XY}\\ T_{A,Y} \end{array}\right]=\left[\begin{array}{cc} \cos^{2}\varphi & \sin^{2}\varphi \\ -2\cos\varphi \cdot \sin\varphi & 2\cos\varphi \cdot \sin\varphi \\ \sin^{2}\varphi & \cos^{2}\varphi \end{array}\right]\left[\begin{array}{c} T_{B,H}\\ T_{B,V} \end{array}\right]$$

where, $T_{B,H}$ and $T_{B,V}$ are the TB in the earth reference frame in H-pol and V-pol, respectively. $T_{A,X}$, $T_{A,Y}$ and $T_{A,XY}$ are the TB in the antenna reference frame after rotation. φ is the angle of polarization rotation plus the Faraday rotation.

#### 2.3.2. Image Reconstruction Model

The IMR measures visibilities that are complex cross-correlations between signals collected by pairs of antenna elements located within the receivers installed on the Y-shaped antenna array. The relationship between the visibility function V(u,v) and the simulated TB $T_{B}\left(\xi ,\eta \right)$ in the antenna coordinate, described as direction cosine (ξ,η), can be expressed in Equation (5), considering the weighted function of the antenna pattern [24].

(5) $$V_{i,j}\left(u,v\right)=\iint T_{B}\left(\xi ,\eta \right)\cdot \frac{F_{i}\left(\xi ,\eta \right)F_{j}^{*}\left(\xi ,\eta \right)}{\sqrt{1-\xi^{2}-\eta^{2}}\sqrt{\Omega_{i}\Omega_{j}}}\cdot {\tilde{r}}_{ij}\cdot e^{-j2\pi \left(u\xi +v\eta \right)}d\xi d\eta$$

where the subscripts i and j (i≠j) indicate the antenna pairs, and (u,v) is the baseline and is equal to the difference between the positions of *i*-th and *j*-th antenna element normalized to the wavelength. F(ξ,η) and $F^{*}\left(\xi ,\eta \right)$ are the conjugate pairs of the antenna pattern after normalization. Ω is the solid angle of the antenna. $\tilde{r}$ is the fringe-washing function, which accounts for spatial decorrelation effects and depends on the frequency response of the pairs of elements collecting the signals being correlated.

Then, the simulated TB images can be reconstructed from the visibilities through an inverse Fourier transform of the latter in the ideal case when decorrelation effects are negligible and all the antennas have the same voltage radiation pattern [25]. Due to the characteristic of Y-shaped antenna array, IMR’s TB images are equipped with hexagonal shaped field of view (FOV) on hexagonal pixels with varying TB radiometric resolutions and spatial resolutions.

The TB radiometric resolution, also known as the radiometric sensitivity, is defined as the minimum input change that can be detected by the instrument. In an interferometric radiometer, it is limited by the discretization and the finite coverage of the baseline (u,v) and the signal noise ratio, which can be improved by increasing the integration time and/or the pre-detection bandwidth [26]. The TB radiometric resolution for each pixel in the FOV is approximately computed by Equation (6).

(6) $$\Delta T\left(\xi ,\eta \right)=\Omega \cdot \frac{\sqrt{3}}{2}d^{2}\cdot \frac{T_{sys}}{\sqrt{B\cdot \tau_{eff}}}\cdot \alpha_{w}\cdot \alpha_{ol}\cdot \frac{\sqrt{1-\xi^{2}-\eta^{2}}}{\bar{F}\left(\xi ,\eta \right)}$$

where, $\frac{\sqrt{3}}{2}d^{2}$ is the value for Y-shaped antenna array, and *d* is the minimum spacing distance between antenna elements which is normalized to the wavelength of L-band (*d* = 0.82 for IMR). $T_{sys}$ is the averaged system temperature which is calculated as $T_{sys}=T_{A,\;ave}+T_{R}$, where $T_{A,\;ave}$ is the averaged TB of the simulated scene and $T_{R}$ is the receiver’s noise temperature ($T_{R}=140K$ for the IMR). *B* is the IMR’s bandwidth and $\tau_{eff}=\tau /c_{eff}$ is the effective integration time. $\alpha_{w}=\sqrt{\underset{u}{\sum }\underset{v}{\sum }\left(w\left(u,v\right)\right)/R\left(u,v\right)}$ accounts for the window and redundancies in the measurements, and $\alpha_{ol}$ is the local oscillator factor. $\sqrt{1-\xi^{2}-\eta^{2}}$ is the obliquity factor, and $\bar{F}\left(\xi ,\eta \right)$ is the averaged antenna pattern after normalization.

Regarding the spatial resolution of each pixel in the FOV, the footprint shape on the ground is computed as the projection of the 3dB contour of the synthetic antenna directional gain which is also known as the equivalent array factor [27]. The equivalent array factor of the IMR in the direction $\left(\xi^{\prime },\eta \prime \right)$ can be interpreted by Equation (7).

(7) $$AF\left(\xi ,\eta ,\xi^{\prime },\eta^{\prime }\right)=\frac{\sqrt{3}}{2}d^{2}\underset{m}{\sum }\underset{n}{\sum }w\left(u_{mn},v_{mn}\right)\cdot \tilde{r}\cdot e^{j2\pi \left(u_{mn}\left(\xi -\xi^{\prime }\right)+v_{mn}\left(\eta -\eta \prime \right)\right)}$$

where, the fringe-washing function can be approximated as r˜≈e[−π(Bf0)2(u⋅ξ+v⋅η)2], and $f_{0}$ is the central frequency of the IMR. The window function used in this simulation is the Blackman window as expressed in Equation (8)

(8) $$W\left(u,v\right)=0.42+0.5\cdot cos\left(\pi \frac{\sqrt{u^{2}+v^{2}}}{\sqrt{3}N_{EL}d}\right)+0.08\cdot cos\left(2\pi \frac{\sqrt{u^{2}+v^{2}}}{\sqrt{3}N_{EL}d}\right)$$

where, $N_{EL}$ is the number of the antenna elements in each arm.

Outputs of the image reconstruction model are the simulated TB images that are derived from the target TB scenes considering the TB radiometric resolution and the spatial resolution according to the configuration of the IMR (Figure 3). Due to the aliasing effect of the interferometric radiometer, TB measurements outside the alias-free domain of the FOV are not suitable to invert SSS accurately. Only TBs belong to the alias-free FOV (AF-FOV) and the extended alias free FOV (EAF-FOV) areas are extracted and used for the SSS retrieval step.

#### 2.3.3. Sea-Surface Salinity (SSS) Retrieval Model

Before parameter retrieval, all the simulated TB images are projected into Earth-fixed grids through the geolocation process. The Equal-Area Scalable Earth (EASE) Grid [28] of 25 km resolution is used in this simulation. As the satellite moves, an earth grid is imaged multiple times by successive TB images. Consequently, each retrieval grid can be featured with TB measurements at different incidence angles, having different TB radiometric resolutions and footprints (spatial resolutions).

The final step is to derive parameter SSS from a set of TB measurements for each Earth-fixed grid. A maximum-likelihood Bayesian approach is used, considering the priori information of SSS. The retrieval is based on the nonlinear iterative convergence algorithm named Levenberg–Marquard (LM) algorithm [29]. Through the minimization of the constrained cost function, we can find the solutions of the aimed parameter, SSS. The constrained cost function F for the retrieval grid is expressed in Equation (9).

(9) $$F\left(SSS\right)=\overset{M}{\underset{i=1}{\sum }}\frac{\left(Tb_{meas}\left(\theta ,p\right)-Tb_{model}\left(\theta ,p\right)\right)}{\sigma^{2}}+\frac{SSS-SSS_{prior}}{\sigma_{SSS}^{2}}$$

where, $Tb_{meas}$ indicate TB measurements in the earth fixed grid with different incidence angles θ, p indicated the H-pol and V-pol, and $Tb_{mod}$ are the corresponding simulated TBs calculated with the TB forward model. $\sigma^{2}=\sigma^{2}{}_{meas}+\sigma^{2}{}_{model}$ account for both the instrument noise $\sigma^{2}{}_{meas}$ described as the TB radiometric resolution and the model error $\sigma^{2}{}_{model}$. M indicates the number of TB measurements. $SSS_{prior}$ is the priori estimate of SSS with a priori variance $\sigma_{SSS}^{2}$.

## 3. Results

Results of the IMR’s antenna array orientation performance based on the end-to-end simulation model are presented in this section. The results consist of simulated TB images, incidence angles, TB radiometric resolutions, spatial resolutions, numbers of the measurements in the Earth grid, SSS accuracy and sun effect.

### 3.1. Simulated TB Image

**Figure 4:**
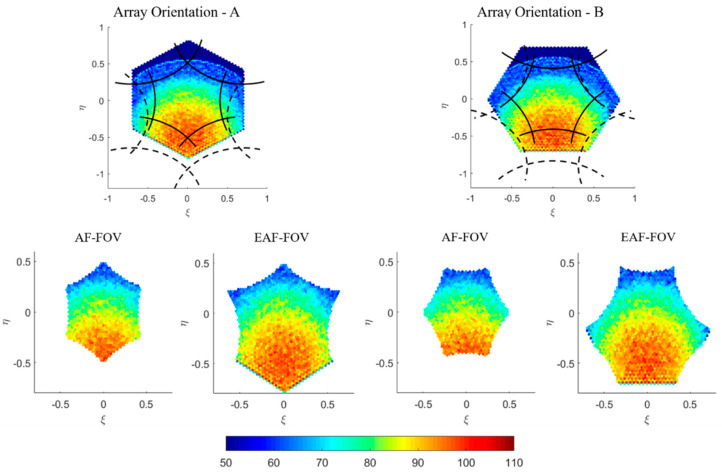
Illustration of the hexagonal-shaped brightness temperature (TB) image in horizontal polarization observed by the IMR in the antenna coordinate, according to different array orientations. The whole field of view (FOV) images are shown in the top, where the black solid lines represent for the six replicas (aliases) of the unit circle and the black dashed lines represent for the periodic repetition of the earth’s disk. TB images in the alias-free FOV (AF-FOV) and extended alias-free FOV (EAF-FOV) areas extracted from the whole FOV are displayed in the bottom. The axes are the director cosines (ξ−η) and the color bar represents TB values in Kelvin.

Due to the characteristic of the 2D interferometric radiometer with the Y-shaped array structure, the IMR has hexagonally sampled scenes with hexagonal-shaped grids. Figure 4 presents the simulated TB images for two different array orientations which are both hexagonal-shaped but have different placement in (ξ,η) coordinate (in accordance with the array orientation, which is 90° clockwise from A to B).

The measurement in the AF-FOV is limited by the periodic repetition of the unit circle (inner region of solid lines in Figure 4). However, since a significant part of the aliases corresponds to cold sky, the AF-FOV can be extended to the EAF-FOV which is up to the region limited by the periodic repetition of the Earth’s disk (inner region of dashed lines in Figure 4). Then TB images in the AF-FOV and EAF-FOV are extracted from the original images which are displayed in the bottom row of Figure 4, respectively. Figure 4 presents TB images in H-pol. Although IMR is a full-polarization radiometer measuring TB of H-pol, V-pol and T3 simultaneously, T3 are mainly used for the correction of Faraday rotation at L-band. Only TB in H-pol and V-pol are used in the following SSS retrieval and errors due to the Faraday rotation are not considered.

### 3.2. Incidence Angle

**Figure 5:**
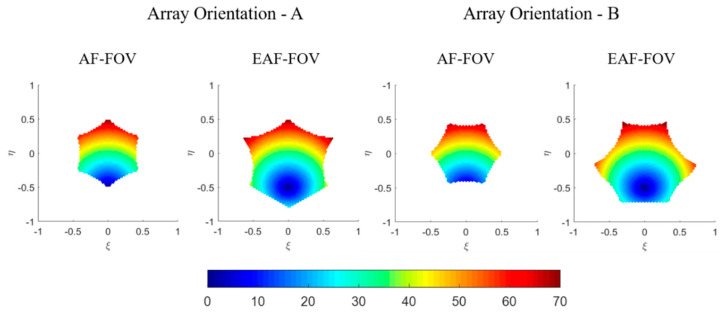
Illustration of the local incidence angle distributions in the antenna coordinate, according to different array orientations. The distributions in the AF-FOV and EAF-FOV are shown separately. The axes are the director cosines (ξ−η) and the color scale is in degrees.

Figure 5 illustrates the distribution of local incidence angles for array orientations A and B, in the AF-FOV and EAF-FOV, respectively. They all display in ξ−η coordinate in accordance with the simulated TB images in Figure 4.

The distribution and the variation range of the incidence angle for array orientations A and B are similar. With respect to the array orientation A, the variation ranges of the incidence angle are from 1.5° to 70° for the AF-FOV and from 0° to 70° for the EAF-FOV. For the array orientation B, the incidence angle varies from about 7° to 67° for the AF-FOV and from 0° to 72° for the EAF-FOV.

### 3.3. TB Radiommetric Resolution

**Figure 6:**
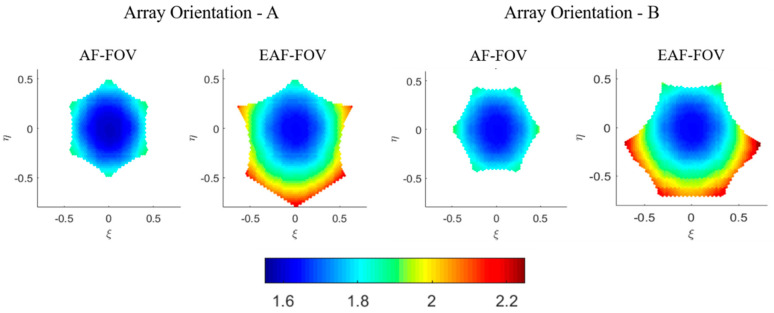
Illustration of the TB radiometric resolution (radiometric sensitivity) distributions of horizontal polarization in the antenna coordinate, according to different array orientations. The distributions in the AF-FOV and EAF-FOV are shown separately. The axes are the director cosines (ξ−η) and the color scale is in Kelvin.

The H-pol TB radiometric resolutions (radiometric sensitivity) in the AF-FOV and EAF-FOV for array orientations A and B are shown in Figure 6, for example. The averaged TB of the simulated scenes used to calculate TB radiometric resolutions are set to 80 K which is a typical value for the ocean scene in H-pol.

The TB radiometric resolution at the boresight is independent of the array orientation and is only related to parameters like the antenna minimum spacing, the bandwidth, the integral time etc. Therefore, values of TB radiometric resolutions at the boresight for array orientations A and B are the same, which are 1.6 K with respect to the typical ocean scene. As can be seen from each subplot in Figure 6, TB radiometric resolutions achieve the optimal value at the boresight, and worsen while approaching the FOV edge. Regarding the AF-FOV for both array orientations A and B, the variation ranges of TB radiometric resolutions are almost the same with the variation range from 1.6 to 1.9 K. Even for the EAF-FOV, the difference of the variation ranges is small, which varies from 1.6 to 2.2 K with respect to the array orientation A, and varies from 1.6 to 2.3 K with respect to the array orientation B.

### 3.4. Spatial Resolution

**Figure 7:**
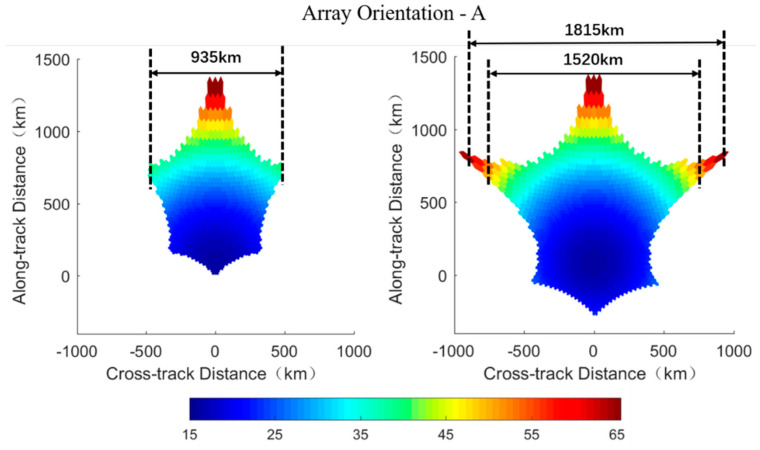
Illustration of the ground spatial resolution distributions of the array orientation A. The distributions in the AF-FOV and EAF-FOV are shown in the left and right panels, respectively, and the corresponding swath widths are labeled in each subplot. The color bar represents for the square root of each pixel’s area in km.

**Figure 8:**
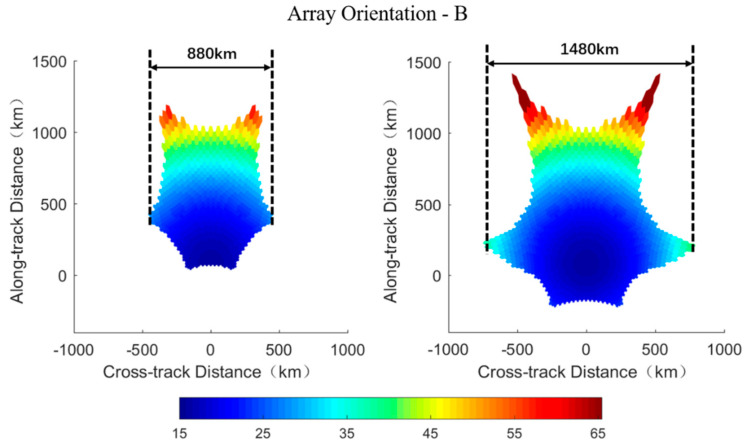
Illustration of the ground spatial resolution distributions of the array orientation B. The distributions in the AF-FOV and EAF-FOV are shown in the left and right panels, respectively, and the corresponding swath widths are labeled in each subplot. The color bar represents the square root of each pixel’s area in km.

The ground spatial resolution of each footprint is calculated from the equivalent array factor expressed in Equation (7) according to the parameters of the radiometer and the orbit. Simulation results of footprints’ shape in the AF-FOV (left columns) and the EAF-FOV (right columns) for array orientations A and B are presented in Figure 7 and Figure 8, respectively. The specific values of ground spatial resolutions shown in Figure 7 and Figure 8 are computed according to the square root of each footprint’s area. As can be seen from the figures, the spatial resolution is illustrated in the ordinate where the *x*-axis represents for cross-track distance and the *y*-axis represents for the along-track distance, and the footprint at boresight locates at the ordinate origin. The swath width measured along the track is estimated based on the results of the ground spatial resolution and labeled in Figure 7 and Figure 8.

Array orientations have more impact on the ground spatial resolution than on the TB radiometric resolution. The ground spatial resolution achieves optimum values at boresight and worsen while approaching the FOV edge, which is similar to the distribution of TB radiometric resolutions (Figure 7 and Figure 8). Meanwhile there exist a trade-off relationship between the TB radiometric resolution and the spatial resolution. With respect to the array orientation A, the ground spatial resolution varies from about 15 km at boresight to about 65 km at edge for both AF-FOV and EAF-FOV. As for the array orientation B, the variation range of the spatial resolution is from about 15 km to about 55 km for the AF-FOV and from about 15 km to about 75 km for the EAF-FOV.

The array orientation A results in a wider swath than the array orientation B for both AF-FOV and EAF-FOV (Figure 7 and Figure 8). The swath width is about 935 km and about 880 km for array orientations A and B for the AF-FOV, respectively. Regarding the EAF-FOV, the swath width extends to about 1815 km (about 1520 km, if considering footprints better than 50 km only) and about 1480 km for array orientations A and B, respectively.

### 3.5. Number of Measurements in Earth Grid

Before the step of SSS retrieval, all TB images simulated along the orbit need to be projected into a pre-defined fixed grid on the Earth. As the satellite is moving along the orbit, footprints with different incidence angles, TB radiometric resolutions and spatial resolutions from different TB images are projected into one Earth grid. In this section, numbers of the measurements for a single pass in earth grids are counted and the 25 km EASE gird is used in our simulation.

**Figure 9:**
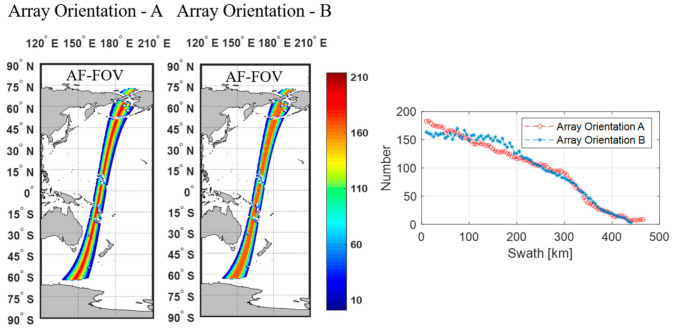
Illustration of the numbers of the repeated measurements in 25 km EASE grid when using the AF-FOV for simulation. The color maps in the left present the distributions of the numbers measured along orbit for two different array orientation. The plots in the right show the variations of the numbers versus the distance to the swath center for two different array orientations.

**Figure 10:**
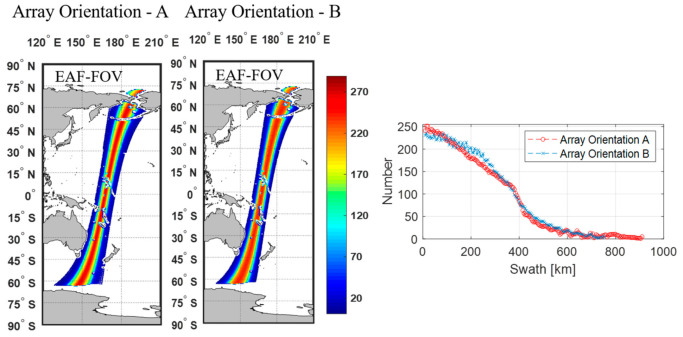
Illustration of the numbers of the repeated measurements in 25 km EASE grid when using the EAF-FOV for simulation. The color maps in the left present the distributions of the numbers measured along orbit for two different array orientation. The plots in the right show the variations of the numbers versus the distance to the swath center for two different array orientations.

Figure 9 and Figure 10 present the number of the repeated measurements for a single pass in the 25 km EASE grid for the array orientations A and B in the AF-FOV and EAF-FOV, respectively.

The largest number of measurements appears in the swath center which reaches 214 if the measurements in the AF-FOV are considered and reaches 290 if the measurements in the EAF-FOV are considered. Then the numbers decrease with the increase of the distance to the swath center for both array orientations. In the circumstance of nearing the swath center, the array orientation A have more measurements than the array orientation B whatever in the AF-FOV or EAF-FOV. The decreasing trend of the array orientation A is flatter than that of the array orientation B within 200 km from the swath center. The plots of numbers versus swath distance also show that the array orientation A achieves better swath width than the array orientation B which is consistent with the results shown in Figure 7 and Figure 8.

### 3.6. SSS Accuracy

Then, SSS values in Earth grids are derived from the SSS retrieval model using the simulated measured TB data after grid projection. The SSS accuracy is analyzed by comparing the retrieved SSS to the Argo SSS which used as the initial field for simulation. The simulation is conducted for two cases: case 1 is an ideal situation assuming no errors in SST and WS, and case 2 considers errors of 1 °C n SST and 2 m/s in WS. The results of SSS accuracy using TB data in the AF-FOV and in the EAF-FOV for array orientations A and B with different error configurations are then analyzed.

**Figure 11:**
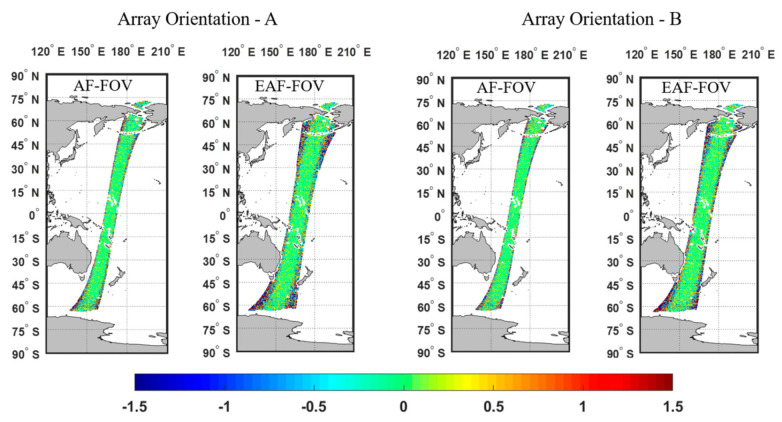
Illustration of the distributions of the SSS errors along one pass, considering the ideal case 1, i.e., with no errors in sea surface temperature (SST) and wind speed (WS). The results of array orientations A and B using TBs in the AF-FOV and in the EAF-FOV are presented, respectively. The color bar represents for the SSS retrieval errors in unit of psu.

**Figure 12:**
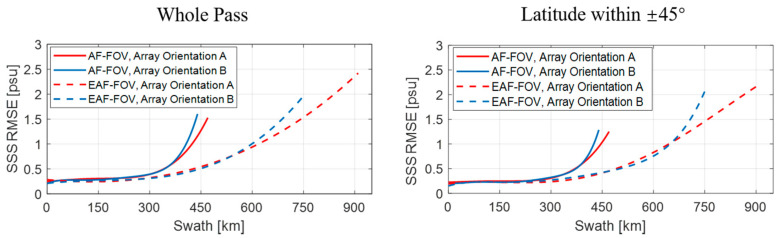
Illustration of the fitting results of the SSS root mean square errors (RMSE) variation versus the distance to the swath center, considering the ideal case 1, i.e., with no errors in SST and WS. The results of array orientations A and B are shown as red and blue lines, and the results for the AF-FOV and EAF-FOV are shown as solid and dashed lines, respectively. The subplots in the left and right panels present the results of the whole pass and of part of the pass with latitude within ±45°.

**Figure 13:**
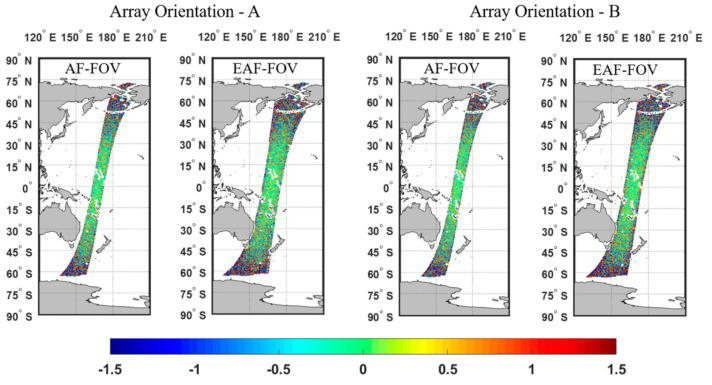
Illustration of the distribution of the SSS errors along one pass, considering case 2, i.e., of 1 °C. SST error and 2 m/s WS error in the SSS retrieval. The results of array orientation A and B with respect to the AF-FOV and EAF-FOV are presented separately. The color bar represents for the SSS retrieval errors in unit of psu.

**Figure 14:**
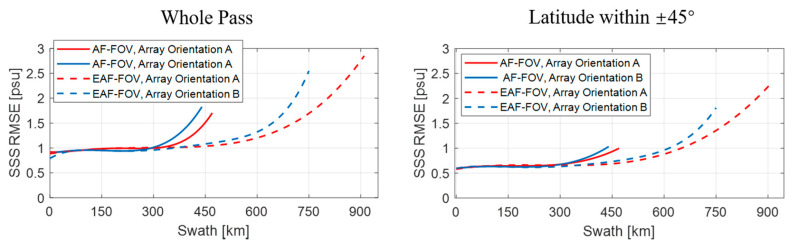
Illustration of the fitting results of the SSS root mean square errors (RMSE) variation versus the distance to the swath center, considering case 2, i.e., of 1 °C. SST error and 2 m/s WS error in the SSS retrieval. The results of array orientations A and B are shown as red and blue lines, and the results for the AF-FOV and EAF-FOV are shown as solid and dashed lines, respectively. The subplots in the left and right panels present the results of the whole pass and of part of the pass with latitude within ±45°.

Figure 11 and Figure 12 present results of the ideal case assuming no errors in SST and WS, i.e., case 1. Figure 13 and Figure 14 present results of case 2. The results of the whole pass and of part of the pass with the latitude within ±45° are presented respectively, since large SSS retrieval errors in high-latitude areas are more likely induced by the low TB to SSS sensitivity in the low SST.

The statistics of SSS retrieval accuracy of the whole pass and of part of the pass with latitude within ±45° are shown in Table 2 and Table 3, including the number of SSS samples (N), the mean value of the retrieval errors (mean) and the root mean square errors (RMSE). The statistics for two different array orientations are calculated with SSS within the same swath widths, i.e., that of the array orientation B (880 km for the AF-FOV, 1480 km for the EAF-FOV).

As can be seen from simulation results of case 1, similar distributions of SSS retrieval errors appear regardless of the array orientation, where most of SSS retrieval errors are within ±0.2psu at the swath center and errors arise when approaching the swath edge (Figure 11). Compared with the SSS using only TBs in the AF-FOV, the SSS using TBs in the EAF-FOV results in larger errors at the swath edge. In general, the SSS RMSE for the whole pass shown in Table 2 increase from about 0.5 psu to about 0.8 psu due to the poor TB radiometric resolution and the smaller number of the measurement at the swath edge.

In Figure 12, the SSS RMSE present similar rising trends with the increase of the distance from the swath center, and the rising trends all become steeper when approaching the swath edge. The SSS RMSE are smaller when applying the EAF-FOV instead of the AF-FOV for both array orientation A and B within the distance of 450 km. Meanwhile, the SSS RMSE are also smaller when considering data with latitude within ±45° only. Two array orientations present consistent results closed to the swath center (within the distance of about 350 km for the SSS using TBs in the AF-FOV and about 600 km for the SSS using TBs in EAF-FOV), but the array orientation A achieves better SSS accuracy than the array orientation B in the swath edge which limits to the swath width of the array orientation B.

In Figure 13 and Figure 14, The results of the case 2 present similar variations but larger SSS RMSE compared with the results of case 1, especially in areas near the poles. For both array orientations A and B, the SSS RMSE for the whole pass shown in Table 2 increase to about 1.0 psu and 1.1 psu for the SSS using TBs in the AF-FOV and in EAF-FOV, respectively. Large errors exist especially near the poles, since the low SST in the poles lead to the low sensitivity of TB to SSS. The results in Figure 14 present similar results compared with the results in Figure 12, that the array orientation A has better SSS accuracy than the array orientation B when the distance from the swath center exceeds 350 km.

The statistics in Table 2 and Table 3 illustrate that the SSS RMSE for the array orientation A is better than the array orientation B considering data within the same swath width of the array orientation B, for both the SSS using TBs in the AF-FOV and in the EAF-FOV, for both case 1 and case 2. The SSS accuracy are much higher concerning for the results within latitude of ±45°, since large SSS errors introduced by low SST in the poles are excluded.

The estimations of the monthly SSS accuracy are illustrated in Table 4 according to the SSS results from case 2, i.e., with errors of 1 °C in SST error and 2 m/s in WS and using TBs in the EAF-FOV, which is close to the realistic situations. The monthly SSS accuracy after applying spatial-temporal averaging depends on the numbers of passes in the monthly averaged grid of 100 km × 100 km and 200 km × 200 km, since more independent observations can reduce random errors raised by the TB radiometric resolution, the instrumental instability, model uncertainties etc. in the SSS retrieval. In the total 450 passes for 30 days, the numbers of the independent passes for the monthly averaging grid are estimated as $N_{pass}$ (Table 4), according to the orbit configuration shown in Section 2.3 for different array orientations. As can be seen from Table 4, the array orientation A with the wider swath leads to more pass coverages. With the more pass coverages and the slightly higher single-pass accuracy than the array orientation B, the array orientation A achieves better monthly SSS accuracies which are below 0.09 psu for both grid of 100 km × 100 km and 200 km × 200 km (Table 4).

### 3.7. Sun Effect

At L-band, the Sun is indeed an extremely strong and time-dependent source and raises a significant challenge for the remote sensing of SSS [5]. There are two distinct ways that the Sun can contaminate measurements of an interferometric radiometer i.e., the Sun’s direct radiation into the antenna, and the reflected sun radiation by the sea surface (namely sun glint or indirect effect). The direct and reflected Sun effects in SMOS with a 2D interferometric radiometer MIRAS have been studied and proven to be significant, even after applying techniques to reduce these effects during the image reconstruction process [30].

**Figure 15:**
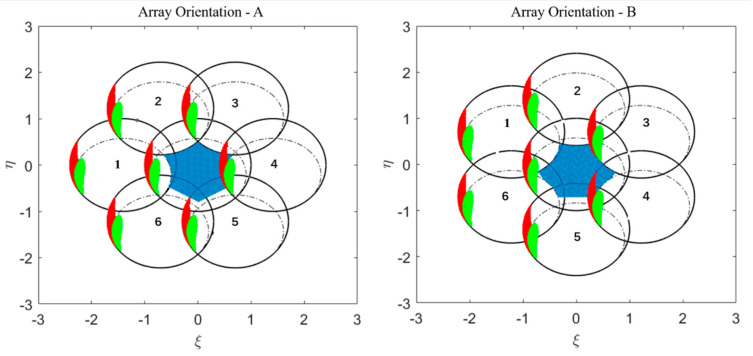
Illustration of the geometric place of the Sun’s positions in the IMR’s main sampling period and the corresponding six closest replicas, where the direct and reflected Sun are shown as red and green images, respectively. The black solid lines represent for the six replicas of the unit circle numbered as 1~6, and the black dashed lines are for the periodic repetition of the Earth’s disk. The EAF-FOV is labeled as blue images.

According to the orbit of the Chinese Ocean Salinity Satellite, a Sun-synchronous orbit with a local equator crossing time at 6 a.m. on the descending node, the Sun always locates at the left side of the FOV (which is just the opposite SMOS [31]). Figure 15 illustrates the Sun’s effects for a year through aliasing based on the orbit and characteristics of the IMR with respect to two different array orientations. From the relative position of the direct (red images) and the reflected Sun (green images) to the EAF-FOV (blue images), the Sun exerts influence on TB measurements in the EAF-FOV through the replicas of the main period. For the array orientation A, the Sun affects the measurement in the right side of the EAF-FOV through the 4th replica. For the array orientation B, the measurement of the EAF-FOV will be contaminated by the Sun through both the 3th and 4th replicas.

## 4. Conclusions and Discussion

This paper analyzes the array orientation performance of the payload IMR onboard Chinese Ocean Salinity Satellite, which is an L-band, two-dimensional interferometric radiometer with a Y-shaped antenna array. The analysis is accomplished by applying two different array orientations to the observation of SSS through an end-to-end simulation system based on the configuration of the IMR.

Several factors that can influence the measurement of SSS with respect to the two array orientations of the IMR are simulated and analyzed. The different orientations result in different forms of hexagonal-shaped TB images observed by the Y-shaped antenna array, that further lead to differences in distributions of incidence angles, TB radiometric resolutions and spatial resolutions. The variation ranges of the incidence angles and the TB radiometric resolutions are similar for two different array orientations, and their differences only reflect in the distributions which are in accordance with their simulated TB images. With respect to the EAF-FOV, the difference in the spatial resolutions are obvious. The array orientation A has better spatial resolutions varying from about 15 to about 65 km compared with those from about 15 to 75 km for the array orientation B. Meanwhile, the effective swath width for the array orientation A is about 1815 km which is also wider than that of about 1480 km for the array orientation B.

Numbers of measurements in the 25 km EASE grid for two different array orientations present a similar decreasing trend versus the distance from the swath center. The array orientation A has a larger amount of the measurements in the swath center than the array orientation B. The retrieved SSS errors present similar distribution despite the array orientations. Large errors mainly locate at the swath edge and the areas close to the poles. But the SSS RMSE versus the distance from the swath center show that the array orientation A has better performance when approaching the swath edge. According to the results of the SSS RMSE for the whole pass and for part of the pass with latitude within ±45°, the array orientation A achieves better accuracy than the array orientation B. However, SSS using TBs in the EAF-FOV can contribute to the monthly average of SSS. Due to the wider swath width and the better accuracy of a single pass, the array orientation A also has better monthly SSS performance which can produce accuracy better than 0.09 psu. A strong external contamination source, the Sun, is also analyzed and the differences of the influence for two different array orientations are presented.

Regarding the choice of the array orientation for the payload IMR onboard the Chinese Ocean Salinity Satellite, array orientation A has better performance than array orientation B. Compared with array orientation B, array orientation A can give a wider swath, a better SSS accuracy at the swath edge and a better monthly averaged SSS. A wider swath means more data can be involved in the monthly averaging, and the better data quality at the swath edge will also result in higher accuracy for the monthly SSS product. The simulations in this paper provide guidance for the selection of the optimal antenna array orientation and then help the hardware design of the payload IMR onboard the Chinese Ocean Salinity Satellite, which will be launched around 2022.

## Figures and Tables

**Table 1:** Comparison between IMR and Soil Moisture Ocean Salinity (SMOS) mission’s Microwave Imaging Radiometer with Aperture Synthesis (MIRAS).

Index	IMR	MIRAS
Frequency (GHz)	1.4	1.4
Bandwidth (MHz)	20	27
Integral Time (s)	0.9	1.2
Number of Elements	56	69
Antenna Spacing	$0.82\lambda_{L}$	$0.875\lambda_{L}$
Spatial Resolution (km)	~35	~50
Radiometric Resolution at Boresight for Ocean (K)	~1.5	~2.5
Polarization	H, V, T3 Simultaneously	HHH, VVV, HHV or VVH One mode per measurement

**Table 2:** The statistics of SSS retrieval accuracy of the whole pass for different array orientations of case 1 and case 2.

Whole Orbit		Case 1 $(\sigma_{SST}=0$$,\;\sigma_{WS}=0)$		Case 2 $(\sigma_{SST}=1\;{}^{\circ}C$$,\;\sigma_{WS}=2\;m/s)$	
		AF-FOV	EAF-FOV	AF-FOV	EAF-FOV
Array Orientation A	N	32,951	52,122	32,906	52,047
	mean	0	0	0.042	0.045
	RMSE	0.509	0.737	1.037	1.129
Array Orientation B	N	33,389	53,849	33,344	53,747
	mean	0	0	0.045	0.043
	RMSE	0.563	0.759	1.069	1.182

**Table 3:** The statistics of SSS retrieval accuracy of part of the pass with latitude with ±45° for different array orientations of case 1 and case 2.

Latitude within ± 45°		Case 1 $(\sigma_{SST}=0$$,\;\sigma_{WS}=0)$		Case 2 $(\sigma_{SST}=1\;{}^{\circ}C$$,\;\sigma_{WS}=2\;m/s)$	
		AF-FOV	EAF-FOV	AF-FOV	EAF-FOV
Array Orientation A	N	22,562	36,163	22,517	36,088
	mean	0	0	0.041	0.038
	RMSE	0.380	0.551	0.685	0.768
Array Orientation B	N	22,888	37,333	22,843	37,231
	mean	0	0	0.047	0.038
	RMSE	0.420	0.600	0.713	0.837

**Table 4:** Estimation of monthly SSS accuracy for different array orientations.

	Array Orientation A		Array Orientation B	
$SSS_{pass}$, EAF-FOV, Case 2	1.1 psu		1.2 psu	
	$N_{pass}$	$SSS_{Monthly}$	$N_{pass}$	$SSS_{Monthly}$
100 km × 100 km	~159	0.087 psu	~142	0.101 psu
200 km × 200 km	~165	0.085 psu	~148	0.099 psu
